# A novel mitochondria-related algorithm for predicting the survival outcomes and drug sensitivity of patients with lung adenocarcinoma

**DOI:** 10.3389/fmolb.2024.1397281

**Published:** 2024-08-08

**Authors:** Xianqiao Wu, Hang Chen, Zhen Ge, Binyu Luo, Hanbo Pan, Yiming Shen, Zuorun Xie, Chengwei Zhou

**Affiliations:** ^1^ Department of Thoracic Surgery, The First Affiliated Hospital of Ningbo University, Ningbo, Zhejiang, China; ^2^ Department of Thoracic Surgery, Ningbo Medical Center LiHuiLi Hospital, Ningbo, Zhejiang, China; ^3^ Shanghai Lung Cancer Center, Shanghai Chest Hospital, Shanghai Jiao Tong University School of Medicine, Shanghai, China; ^4^ Department of Otology and Skull Base Surgery, Eye Ear Nose and Throat Hospital, Fudan University, Shanghai, China

**Keywords:** mitochondria, tumor immunity, consensus cluster, risk assessment model, lung adenocarcinoma

## Abstract

**Background:**

Mitochondria have always been considered too be closely related to the occurrence and development of malignant tumors. However, the bioinformatic analysis of mitochondria in lung adenocarcinoma (LUAD) has not been reported yet.

**Methods:**

In the present study, we constructed a novel and reliable algorithm, comprising a consensus cluster analysis and risk assessment model, to predict the survival outcomes and tumor immunity for patients with terminal LUAD.

**Results:**

Patients with LUAD were classified into three clusters, and patients in cluster 1 exhibited the best survival outcomes. The patients in cluster 3 had the highest expression of *PDL1* (encoding programmed cell death 1 ligand 11) and *HAVCR2* (encoding Hepatitis A virus cellular receptor 2), and the highest tumor mutation burden (TMB). In the risk assessment model, patients in the low-risk group tended to have a significantly better survival outcome. Furthermore, the risk score combined with stage could act as a reliable independent prognostic indicator for patients with LUAD. The prognostic signature is a novel and effective biomarker to select anti-tumor drugs. Low-risk patients tended to have a higher expression of *CTLA4* (encoding cytotoxic T-lymphocyte associated protein 4) and *HAVCR2*. Moreover, patients in the high-risk group were more sensitive to Cisplatin, Docetaxel, Erlotinib, Gemcitabine, and Paclitaxel, while low-risk patients would probably benefit more from Gefitinib.

**Conclusion:**

We constructed a novel and reliable algorithm comprising a consensus cluster analysis and risk assessment model to predict survival outcomes, which functions as a reliable guideline for anti-tumor drug treatment for patients with terminal LUAD.

## 1 Introduction

As the most common malignant tumor worldwide, lung cancer is famous for its high mortality and high heterogeneity among malignant tumors (Oliver, 2022). Lung cancer has shown the highest estimated incidence and mortality in the United States for years (Siegel et al., 2021; Siegel et al., 2022). Similarly, lung cancer has the highest incidence and mortality among patients in China (Xia et al., 2022). As the most common pathological classification of lung cancer, lung adenocarcinoma (LUAD) accounts for approximately 60% of lung cancer, and is considered to be closely related to heredity and gene mutations (Warth et al., 2012). Under appropriate conditions, surgical treatment, especially a video-assisted thoracic surgery, remains the gold standard for the treatment for LUAD, which could dramatically prolong the overall survival (OS) of patients (Merchant et al., 2018). Although the emergence of other therapeutic methods (e.g., molecular targeted therapy and immunotherapy) have improved the life quality of patients with terminal stage lung cancer (Pilotto et al., 2015; Dong et al., 2019), the 5-year survival rate of patients with distant metastasis is only 7% (American Cancer Society, 2024). Therefore, exploring the complex pathogenesis of LUAD and seeking novel and reliable biomarkers are important.

The mitochondrion is a membrane-enclosed structure that produces energy for fundamental cell activities (Tan et al., 2017), which is also involves in hepatic lipid metabolism and oxidative stress (André, 1994; Mansouri et al., 2018). Recently, increasing evidence has demonstrated the crucial role of mitochondria in the occurrence and development of malignant tumors, and mitochondria might be an effective target for patients with cancer (Zhao Y. et al., 2013; Boland et al., 2013; Ubah and Wallace, 2014). For instance, Zhao J. et al. (2013b) proposed that mitochondria could promote the invasion and migration of malignancy by providing a large amount of adenosine triphosphate for pseudopodia. Furthermore, Villa et al. (2017) reported that mitochondria mediated the sensitivity of LUAD cells to chemotherapy drugs by regulating the autophagy signaling pathway. Moreover, Chang et al. revealed that dihydroergotamine tartrate, a drug used to treat migraine, acted on mitochondria in LUAD cells, thereby promoting apoptosis and mitochondrial autophagy (Chang et al., 2016). Thus, mitochondria are involved in the biological behavior of malignant tumor cells, especially LUAD cells.

Mitochondria can not only meet the energy demand of fundamental cellular activities, but also effectively regulate immune activities (Banoth and Cassel, 2018). Porporato et al. proposed that cancer cells could modify the tumor immune microenvironment (TIME) and the immune response of the host by releasing dangerous signals and altering the metabolism of mitochondria (Porporato et al., 2018). Moreover, Klein et al. (2020) reported that mitochondrial oxidative phosphorylation inhibitors targeted cancer-related immune cells in the TIME, and played a crucial part in immune evasion in the occurrence and progression of cancer. Furthermore, Cloonan and Choi (2013) introduced the detailed role of mitochondria as sensors and mediators of innate immune receptor signaling. The precise coordination of oxidative stress between intracellular mitochondria and other organelles is crucial for cell survival. The dynamic balance of oxidative stress can not only coordinate complex cellular signaling events in cancer cells, but also affect other components of the tumor immune microenvironment (TIME). Immune cells, such as M2 macrophages, dendritic cells, and T cells, are the main components of immunosuppressive TMIE induced by oxidative stress (Kuo et al., 2022). Therefore, mitochondria are closely related to the immune activities of cells.

In recent years, immunotherapy has gradually become an effective tumor treatment strategy as an emerging tumor treatment strategy (Ribas and Wolchok, 2018). Unlike traditional radiotherapy and chemotherapy, immunotherapy is a treatment strategy that utilizes the human immune system to attack and eliminate cancer cells. It does not directly destroy tumor cells, but rather activates, enhances, or repairs the patient’s own immune system to recognize and kill tumor cells. The most common immune checkpoint molecules are Programmed Death Ligand 1 (PD-L1) and Hepatitis A Virus Cellular Receiver 2 (HAVCR-2) (Pardoll, 2012). PD-L1 is an immune checkpoint protein that plays an important role in the immunotherapy of malignant tumors. It mainly inhibits T cell activity by binding to the Programmed Cell Death Protein 1 (PD-1) receptor, thereby reducing immune response and helping tumor cells evade immune surveillance. Therefore, inhibiting the interaction between PD-L1 or PD-1 can restore the activity of T cells and enhance the immune killing effect on tumor cells (Herbst et al., 2014; Sunshine and Taube, 2105). HAVCR-2, also known as T-cell Immunoglobulin and Mucin Domain 3 (TIM-3), is another important immune checkpoint molecule. It plays a crucial role in regulating the immune response process, especially in inhibiting T cell function. HAVCR-2 negatively regulates T cell activity by binding to its ligand, such as Galectin-9, and participates in regulating T cell depletion and immune tolerance phenomena. In immunotherapy, inhibition of HAVCR-2 is believed to enhance the anti-tumor effect of T cells, especially in patients who have failed treatment with PD-1/PD-L1 inhibitors and may play an important role. Therefore, HAVCR-2, as a potential target, is actively being studied and developed to expand and enhance the effectiveness of immunotherapy (Sakuishi et al., 2010; Anderson et al., 2016; Das et al., 2017).

The potential relationships among mitochondria, tumor immunity, and LUAD have been reported. For example, Fei et al. (2022) found that mitochondrial topoisomerase I was closely related to immune cells and the expression of immune checkpoint inhibitors (ICIs) in patients with LUAD. However, there has been no bioinformatic study of consensus cluster analysis combined with prognostic signature for patients with LUAD. In addition, mitochondria also participate in the expression of PD-L1 and HAVCR-2 in tumor cells, demonstrating an undeniable role in tumor occurrence and development. The latest research indicates that mitochondria are involved in the localization regulation of PD-L1 protein on the outer membrane and mitochondria. Enhancing mitochondrial autophagy helps to degrade mitochondrial localization PD-L1, thereby overcoming the resistance of TNBC to chemotherapy and immunotherapy (Jabbarzadeh et al., 2020). The research results in this area are of great significance for enhancing the efficacy of targeted PD-1/PD-L1 therapy. In addition, studies have found that mitochondrial autophagy can enhance the therapeutic effect of ICI combined with paclitaxel by degrading mitochondrial distribution PD-L1, which can be inhibited by ATAD3A protein (Xie et al., 2023). Besides, in human colorectal cancer cancer cells, mitochondrial dysfunction inhibits the expression of HAVCR-2, thereby affecting the immune escape of tumor cells (Sakhnevych et al., 2019). Thus, fully understanding the role of mitochondria in the development of LUAD might provide theoretical guidance and new strategies for future mitochondrial targeted therapy.

In the present study, we established a novel and reliable algorithm compromising molecular subtypes and a risk assessment model to predict prognosis and select sensitive anti-tumor drugs for patients with LUAD.

## 2 Materials and methods

### 2.1 Data download

The gene expression at the transcriptome level and corresponding clinical information were downloaded from LUAD project of The Cancer Genome Atlas database (https://portal.gdc.cancer.gov/). Subsequently, the mRNAs and long noncoding RNAs (lncRNAs) were annotated using gene transfer format (GTF) files obtained from Ensembl. A list of mitochondria-related genes (mrgenes) were downloaded from The Gene Ontology Resource (GO, http://geneontology.org/) (The Gene Ontology Consortium, 2019). The mitochondria-related lncRNAs (mrlncRNAs) were identified by performing a Spearman correlation analysis between genes related to mitochondria and lncRNAs (|cor| > 0.4, *P* < 0.001). Differentially expressed mrlncRNAs (DEmrlncRNAs) were filtered using differential expression analysis (|log FC| > 1, false discovery rate <0.001), and DEmrlncRNAs closely related to survival were screened using a univariate Cox analysis (*P* < 0.01), which were visualized using a volcano map and a forest map. We obtained mrlncRNAs that were closely related to the occurrence of LUAD and the OS of patients with LUAD, which were the foundation for the subsequent construction of the consensus cluster analysis and risk assessment model.

### 2.2 Molecular subtypes according to DEmrlncRNAs

The patients with LUAD were classified into different molecular subtypes based on the expression of DEmrlncRNAs by running the ConsensusClusterPlus package (Wilkerson and Hayes, 2010). Then, the survival outcomes of the patients with different molecular subtypes were explored by performing a Kaplan–Meier survival analysis. According to the National Comprehensive Cancer Network guidelines, the expression of common ICIs could reflect the reactivity of patients with LUAD to immunotherapy approximately, which could benefit a large number of patients with a terminal stage tumor (Ettinger et al., 2021). The expression levels of common ICIs (e.g., *PDL1* (encoding programmed cell death 1 ligand 1) and *HAVCR2* [encoding Hepatitis A virus cellular receptor 2)] were compared between patients from different clusters, and a series of boxplots were generated for visualization, which were marked as: ***P < 0.001; **P < 0.01; and *P < 0.05. To better comprehend the relative abundance of stromal cells and immune cells in the TIME, the StromalScore, ImmuneScore, and ESTIMATEScore were calculated using the estimate package, which were subsequently compared between different clusters. The tumor mutation burden (TMB) represents the total number of mutations per million bases, which is used as a rough indicator of the efficacy of immunotherapy (Forde et al., 2018; Anagnostou et al., 2020; Marabelle et al., 2020). Several Wilcoxon rank-sum tests were performed to investigate whether there was a statistical difference in the TMB between patients from different clusters.

### 2.3 Risk assessment model based on DEmrlncRNAs

To better verify the predictive capability of the constructed risk assessment model, the patients with LUAD were equally divided into a training group and a test group randomly. To prevent over-fitting of the constructed model, the Least absolute shrinkage and selection operator regression combined with a multivariate Cox regression analysis were conducted on the DEmrlncRNAs of patients in the training group to construct a novel prognostic signature related to mitochondria. Then, the risk scores of patients in the training group were calculated using the following formula:

$$\text{Risk score}=\sum_{i=1,}^{15}\text{Coef }\left(i\right)\times E\left(i\right),$$

Where E(i) and Coef (i) are the expression and the regression coefficients of the DEmrlncRNAs, respectively. The median value of the risk score for patients in the training group was used as the cut-off point to classify patients into high- and low-risk groups. Subsequently, the regression coefficients and cut-off point of patients in the test group were determined to be in full accordance with those of the patients in train group. Kaplan–Meier survival analyses were conducted to exhibit the survival outcomes of patients in the different risk groups. To evaluate the predictive ability of the risk assessment model, the receiver operating characteristic curves were plotted and the area under the curve was calculated, respectively. To explore the potential relationship between the risk score and survival status, four scatter plots were plotted for visualization. Univariate and multivariate Cox regression analyses were conducted to investigate whether the risk assessment model could function as a reliable independent prognostic indicator for patients with LUAD, which was visualized using four forest maps. Furthermore, to filter the patients with LUAD whose prognosis could be predicted accurately using the risk assessment model, a series of Kaplan–Meier survival analyses were performed for validation. A clinical heatmap was used to exhibit the expression levels of 15 DEmrlncRNAs included in the modeling process, which revealed the potential relationships between the risk group and common clinicopathological characteristics [e.g., node (N), metastasis (M), tumor (T), stage, gender, age, ImmuneScore, and Cluster], in which the clinicopathological characteristics closely related to the risk groups were discussed in detail. Furthermore, to study the response of patients with LUAD to immunotherapy, the TMB and the expression levels of *CTLA4* (encoding cytotoxic T-lymphocyte associated protein 4) and *HAVCR2* in patients in the different risk groups were compared using Wilcoxon rank-sum tests. Moreover, we used the immunophenoscore (IPS), which represents gene expression levels in immune cells closely related to the tumor, including lymphocytes and macrophages, which has been utilized to assess the response to immunotherapy targeting PD-L1 and CTLA-4 (Charoentong et al., 2020). The IPS of each patient was downloaded from The Cancer Immunome Atlas (https://tcia.at/) (Prior et al., 2013), which were compared between different risk groups. The statistical differences in StromalScore, ImmuneScore, and ESTIMATEScore between the different risk groups were explored using Wilcoxon rank-sum tests. Single-sample gene-set enrichment analysis was used to quantify the relative abundance of common immune cells and the relative activity of common immune-related signaling pathways, which were compared between different risk groups. To explore the functions and signaling pathways closely related to the risk groups, two bar-plots were plotted for GO and Kyoto Encyclopedia of Genes and Genomes functional enrichment analyses for visualization. A Sankey diagram was generated to visualize the relationship between the molecular subtypes and the risk assessment model. The half maximal inhibitory concentration (IC_50_) represents the concentration of an anti-tumor drugs that inhibits half of the tumors cells, which could effectively measure the reaction of patients with LUAD to anti-tumor drugs (Sebaugh, 2011). In the present study, the pRRophetic package was run to evaluate the IC_50_ of common anti-tumor drugs including chemotherapy (e.g., Cisplatin, Docetaxel, Gemcitabine, and Paclitaxel) and molecular targeted therapy (e.g., Gefitinib and Erlotinib). A nomogram was plotted to display the calculation process of the risk scores for clinical patients, the predictive capability of which was evaluated using one-, three-, and five-year correction curves. Finally, a survival curve exhibited the survival outcomes of patients from different molecular subtypes and risk groups.

## 3 Results

### 3.1 Identification of DEmrlncRNAs

**FIGURE 1:**
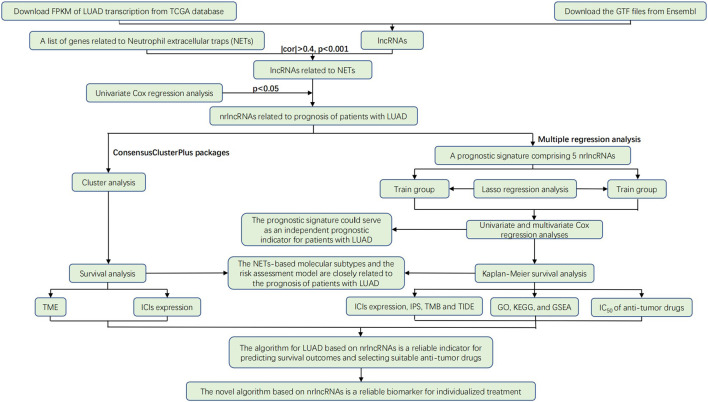
Flowchart showing the steps of this study.

**FIGURE 2:**
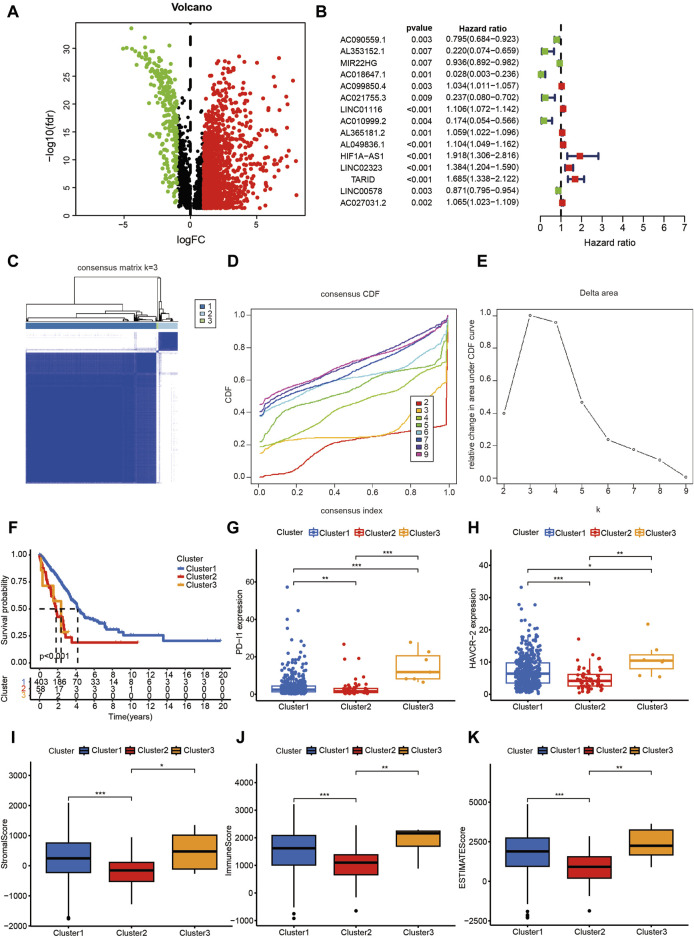
The Consensus cluster analysis based on DEmrlncRNAs. **(A)** 1,724 DEmrlncRNAs were filtered by differential expression analysis, among which 276 showed low expression, and 1,448 showed high expression in patients with LUAD. **(B)** 76 DEmrlncRNAs were considered to be closely related to prognosis using a univariate Cox analysis, 15 of which were included in the modeling process. **(C–E)** When the k value was 3, the variation of cumulative distribution function (CDF) was the smallest, and the relative change in area under the CDF curve was the highest. **(F)** Therefore, the patients with LUAD were classified into three 3 clusters, and patients in cluster 1 exhibited the best survival outcomes, which were statistically significant. **(G, H)** According to the expression of ICIs, the patients in cluster 3 had the highest PD-L1 and HAVCR-2 expression, followed by the patients in cluster 1, and the patients in cluster 2 had the lowest expression, which were all statistically significant. **(I–K)** The patients in cluster 2 had the lowest StromalScore, ImmuneScore, and ESTIMATEScore, which suggested that they had the lowest abundance of stromal cells and immune cells, while there was no statistical significance between the remaining two groups.

As shown in Figure 1, a multi-step approach was carried out according to the flowchart. We downloaded a total of 551 samples (497 LUAD tissues and 54 normal tissues) from The Cancer Genome Atlas database. Then, we downloaded 1,838 mrgenes from the GO knowledgebase, and obtained 14,087 lncRNAs. After annotation, 3,546 lncRNAs were identified as mrlncRNAs by performing correlation analyses. Next, we identified 1,724 DEmrlncRNAs by differential expression analysis, of which 276 were downregulated, and 1,448 were upregulated in patients with LUADs (Figure 2A). Then, 76 DEmrlncRNAs were identified as DEmrlncRNAs closely related to prognosis using a univariate Cox analysis, 15 of which were included in the modeling process (Figure 2B).

### 3.2 The molecular subtype is a reliable indicator for tumor immunity

**FIGURE 3:**
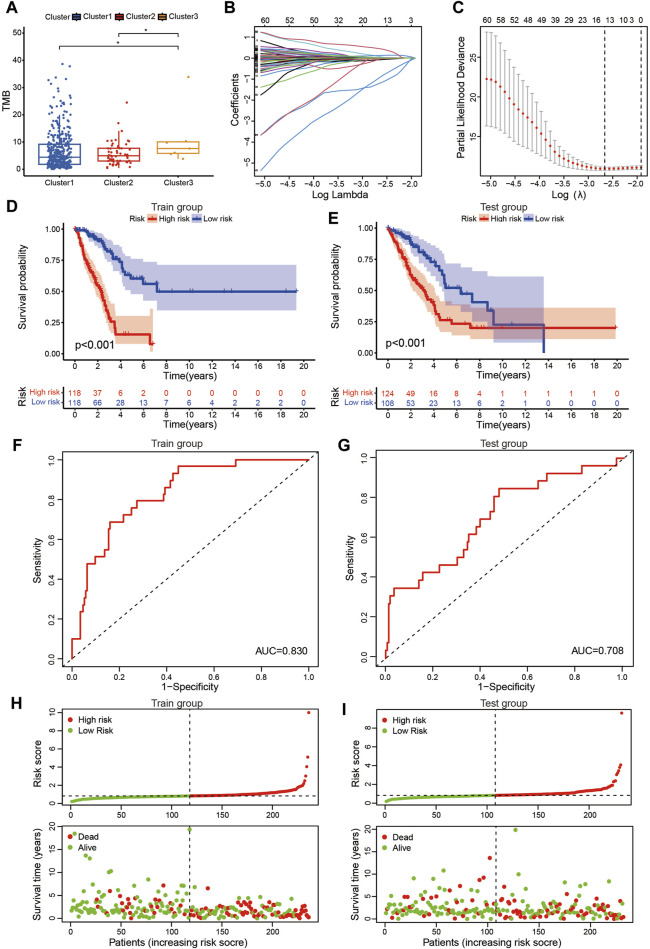
Prognostic signature based on DEmrlncRNAs. **(A)** The patients in cluster 3 had the highest TMB. **(B, C)** 236 Patients were classified into the training group, while 232 patients were arranged into the test group. **(D, E)** The patients in the low-risk group tended to have a significantly better survival outcome. **(F, G)** The area under the curve value of the training group and the test group were 0.830 and 0.708, respectively. **(H, I)** Furthermore, with the accumulation of the risk score, the number of patients who died increased significantly.

When the k value was three, the variation of the cumulative distribution function (CDF) was the smallest, and the relative change in the area under CDF curve was the highest (Figures 2C–E). Therefore, the patients with LUAD were classified into three clusters, and the patients in cluster 1 exhibited the best survival outcomes among the clusters, with statistical significance (Figure 2F). According to the expression of ICIs, the patients in cluster 3 had the highest *PDL1* (Figure 2G) and *HAVCR2* (Figure 2H) expression, followed by the patients in cluster 1; the patients in cluster 2 had the lowest expression, which were all statistically significant. Furthermore, the patients in cluster 3 had the highest TMB (Figure 3A), which indicated that they might be most sensitive to immunotherapy targeting PD-L1 and HAVCR-2. The patients in cluster 2 had the lowest StromalScore, ImmuneScore, and ESTIMATEScore, which suggested that they had the lowest abundance of stromal cells and immune cells, while there was no statistical significance between the remaining two groups (Figures 2I–K).

### 3.3 The prognostic signature acts as a reliable biomarker for patients with LUAD

**FIGURE 4:**
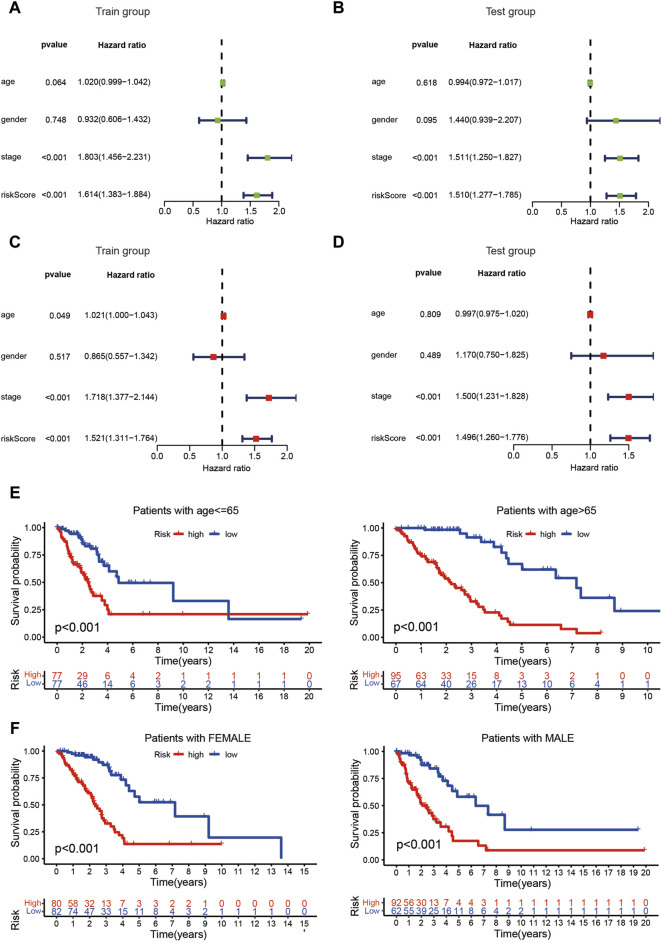
Evaluation of the risk assessment model. **(A–D)** The risk score could act as a reliable independent prognostic indicator for patients with LUAD based on univariate and multivariate Cox regression. **(E, F)** The prognostic signature exhibited the best predictive ability among patients regardless age and sex.

**FIGURE 5:**
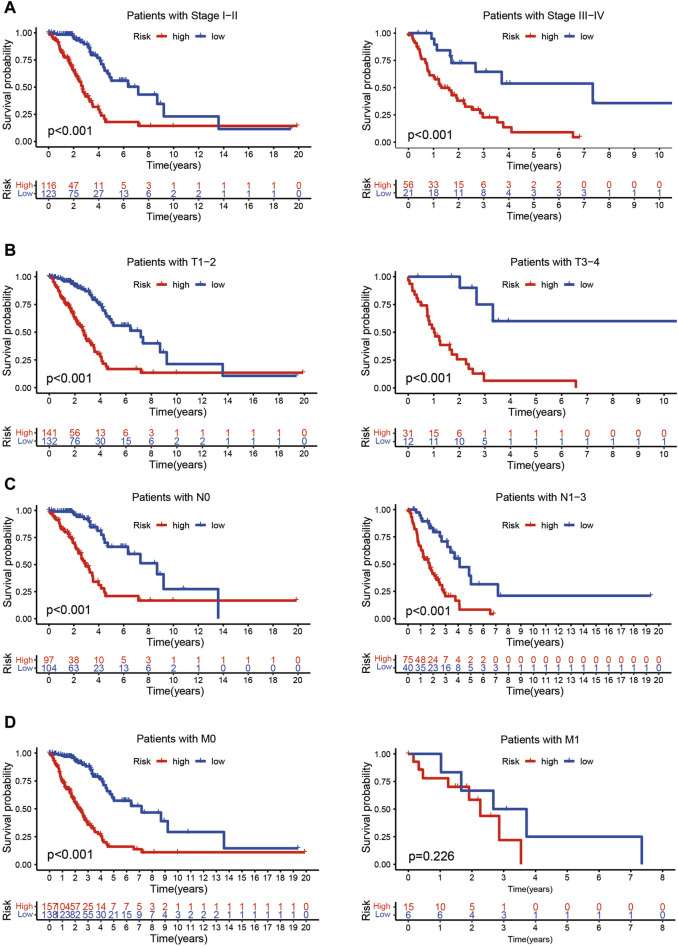
Evaluation of the risk assessment model. **(A–D)** The prognostic signature exhibited the best predictive ability among patients without distant metastasis **(D)**, regardless of stage **(A)**, T **(B)**, or N **(C)** status.

**FIGURE 6:**
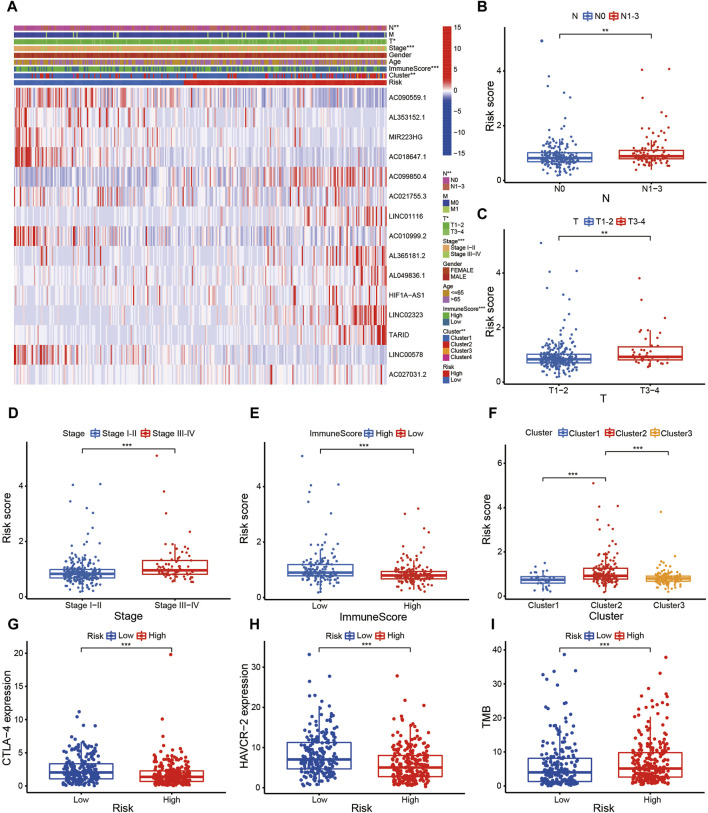
Evaluation of the risk assessment model. **(A–F)** The clinical heatmap **(A)** revealed that the prognostic signature was closely related to N **(B)**, T **(C)**, Stage **(D)**, ImmuneScore **(E)**, and molecular subtypes **(F)**.

The risk score of patients with LUAD were calculated with the formula, and the regression coefficients of the DEmrlncRNAs were listed in Table 1. Patients (n = 236) were classified into the training group, while 232 patients were classified into the test group (Figures 3B, C), in which patients in the low-risk group tended to have a significantly better survival outcome (Figures 3D, E). The area under the curve values of the training group and the test group were 0.830 (Figure 3F) and 0.708 (Figure 3G), respectively, which suggested that the prognostic signature had a relatively better predictive capability for patients with LUAD. Furthermore, with increasing risk score, the number of patients who died increased significantly (Figures 3H, I). Moreover, the risk score (hazard ratio = 1.614 [confidence interval 1.383–1.884], *P* < 0.001) and stage (hazard ratio = 1.803 [confidence interval 1.456–2.231], *P* < 0.001), could act as reliable independent prognostic indicators for patients with LUAD based on univariate and multivariate Cox regression (Figures 4A–D). According to a series of survival analyses, the prognostic signature exhibited the best predictive ability among patients without distant metastasis (Figure 5D), regardless of age (Figure 4E), sex (Figure 4F), stage (Figure 5A), T (Figure 5B), and N (Figure 5C). The clinical heatmap (Figure 6A) revealed that the prognostic signature was closely related to N (Figure 6B), T (Figure 6C), stage (Figure 6D), ImmuneScore (Figure 6E), and molecular subtypes (Figure 6F). This indicated that high-risk patients tended to have a later stage of LUAD and always had relatively poor survival outcomes.

**TABLE 1:** The regression coefficients of mrlnRNAs included in the lasso regression.

Gene	Coef
AC090559.1	−0.0808422085604288
AL353152.1	−0.0128420568313749
MIR223HG	−0.0183021082479998
AC018647.1	−1.20900113082152
AC099850.4	0.011776662913192
AC021755.3	−0.0600844102402713
LINC01116	0.030971078922924
AC010999.2	−0.948844016068309
AL365181.2	0.0130238569092484
AL049836.1	0.0103501622344718
HIF1A-AS1	0.0823378698246388
LINC02323	0.124240788617215
TARID	0.454314559680914
LINC00578	−0.0348236259612791
AC027031.2	0.0131456798726987

**FIGURE 7:**
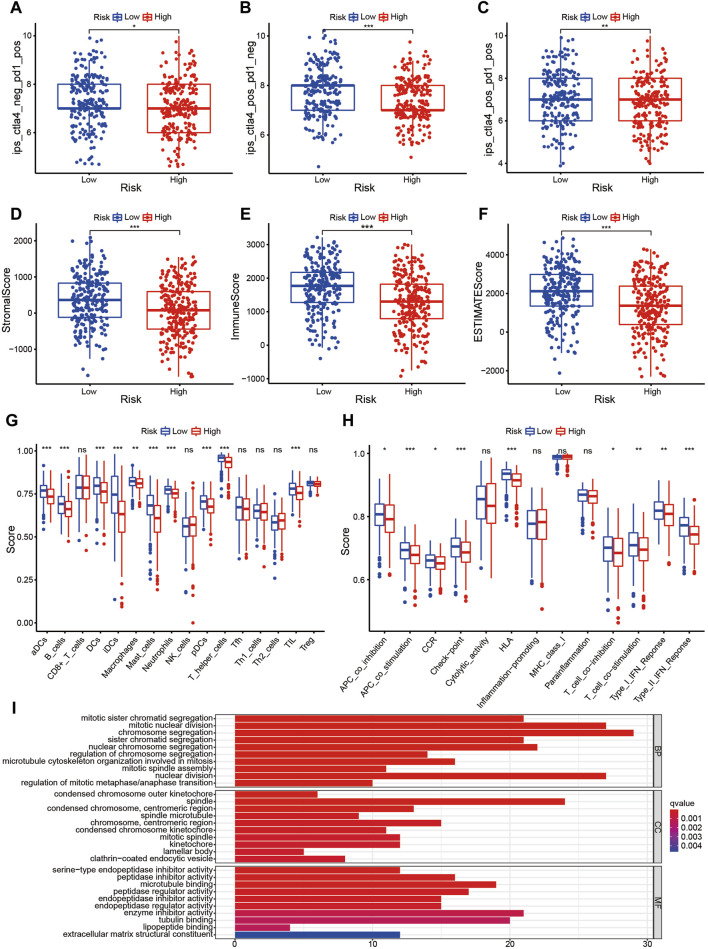
The prognostic signature is a novel and effectively biomarker to select anti-tumor drugs (I). **(A–C)** Low-risk patients always benefitted from anti-PD-L1 therapy **(A)**, anti-CTLA-4 therapy **(B)**, and their combination **(C)**, with statistical significance. **(D–F)** The low-risk patients possessed a higher abundance of stromal cells and immune cells based on the estimate algorithm. **(G, H)** The low-risk patients had a higher content of common immune cells **(G)**, and more active immune-related signaling pathways **(H)**. **(I)** Compared with the high-risk patients, the patients in the low-risk group tended to have stronger tumor immunity.

**FIGURE 8:**
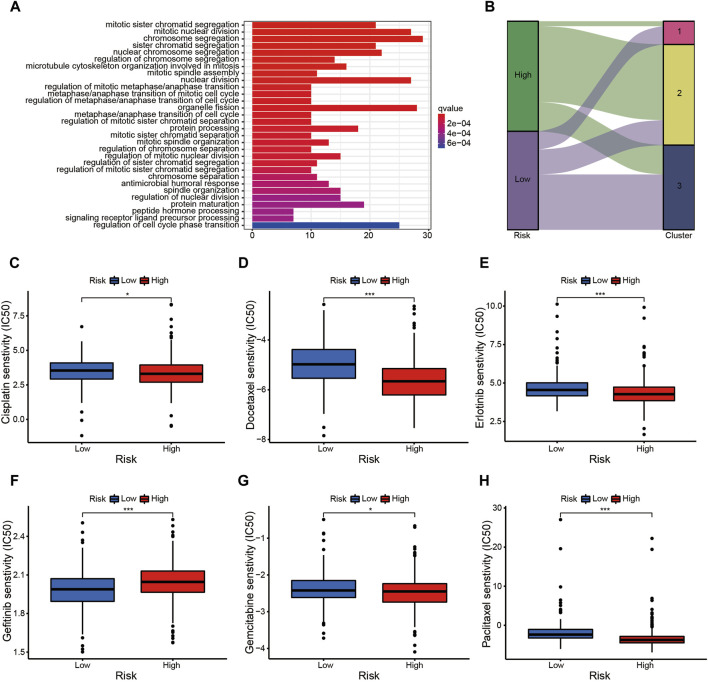
The prognostic signature is a novel and effective biomarker to select anti-tumor drugs (II). **(A)** The mitotic-related signaling pathways were enriched in the risk assessment model, such as mitotic sister chromatid segregation, mitotic nuclear division, chromosome segregation, nuclear division, and organelle fission, etc. **(B)** The majority of high-risk patients were from cluster 2, the most low-risk patients were from cluster 3, while 80% of the patients from cluster 1 were low-risk. **(C–F)** The patients in high-risk group were more sensitive to Cisplatin **(C)**, Docetaxel **(D)**, Erlotinib **(E)**, Gemcitabine **(G)**, and Paclitaxel **(H)**; while the low-risk patients probably benefitted more from Gefitinib **(F)**.

The prognostic signature is a novel and effectively biomarker to select anti-tumor drugs. The low-risk patients tended to have a higher expression of *CTLA4* (Figure 6G) and *HAVCR2* (Figure 6H) with a lower TMB (Figure 6I), suggesting that they might be more sensitive to immunotherapy targeting CTLA-4 and HAVCR-2. According to the IPS value, low-risk patients would always benefit from anti-PD-L1 therapy (Figure 7A), anti-CTLA-4 therapy (Figure 7B), and their combination (Figure 7C), with statistical significance. The low-risk patients possessed a higher abundance of stromal cells and immune cells, based on the estimate algorithm (Figures 7D–F). Furthermore, the low-risk patients had a higher content of common immune cells (Figure 7G) and more active immune-related signaling pathways (Figure 7H). Therefore, compared with the high-risk patients, the patients in low-risk group tended to have stronger tumor immunity. According to the GO functional enrichment analysis, the mitochondria-related signature was closely related to the process of mitosis, including mitotic sister chromatid segregation, mitotic nuclear division, chromosome segregation, and sister chromatid segregation (Figure 7I). Similarly, the mitosis-related signaling pathways were enriched in the risk assessment model, such as mitotic sister chromatid segregation, mitotic nuclear division, chromosome segregation, nuclear division, and organelle fission (Figure 8A). The majority of high-risk patients were from cluster 2, the most of low-risk patients were from cluster 3, while 80% of the patients from cluster 1 were low-risk (Figure 8B).

**FIGURE 9:**
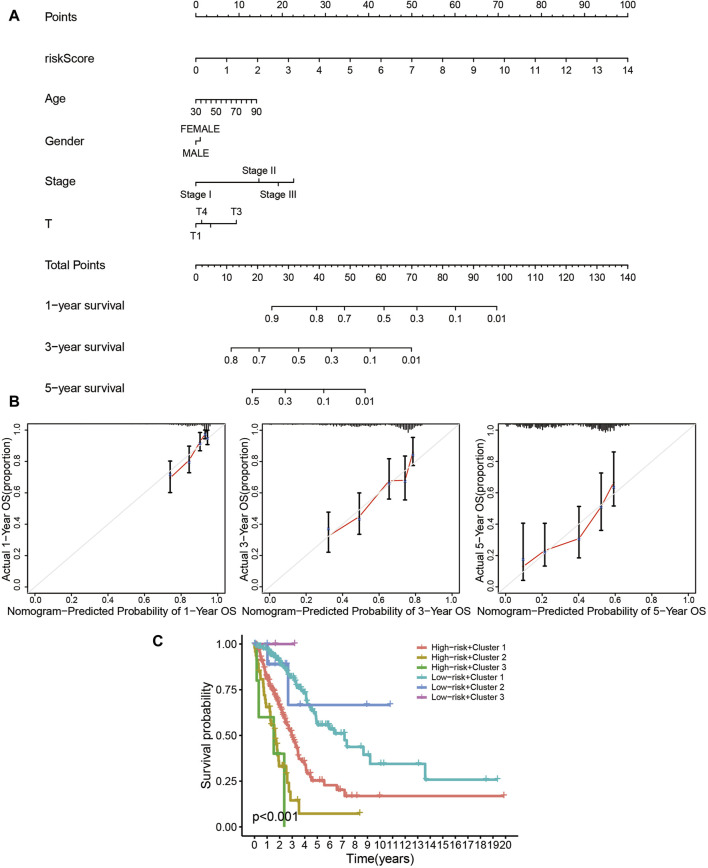
The prognostic signature is a novel and effective biomarker for patient survival. **(A, B)** The nomogram simplified the calculation process of the risk score, and provided the corresponding approximate one-, three-, and five-year survival rate based on the calculated risk score **(A)**, in which the nomogram exhibited the best predictive capability for one-year survival **(B)**. **(C)** According to the multi-survival curve, the high-risk patients in cluster 2 and cluster 3 exhibited poor survival outcomes, while the low-risk patients in cluster 1 showed a relative survival advantage.

The risk assessment model could function as a robust guideline for clinical medication using common chemotherapies and molecular targeted therapy. For example, patients in the high-risk group were more sensitive to Cisplatin (Figure 8C), Docetaxel (Figure 8D), Erlotinib (Figure 8E), Gemcitabine (Figure 8G), and Paclitaxel (Figure 8H), while low-risk patients would probably benefit more from Gefitinib (Figure 8F). The nomogram simplified the calculation process of the risk score, and provided the corresponding approximate one-, three-, and five-year survival rates based on the calculated risk score (Figure 9A), in which the nomogram exhibited the best predictive capability for 1-year survival (Figure 9B). According to the multi-survival curve, the high-risk patients in cluster 2 and cluster 3 exhibited poor survival outcomes, while the low-risk patients in cluster 1 had a relative survival advantage (Figure 9C).

## 4 Discussion

Changes in mitochondrial homeostasis are closely related to many human diseases, such as cancer, neurodegenerative diseases [Parkinson’s disease (Macdonald et al., 2018), Alzheimer’s disease (Swerdlow, 2018), and Huntington’s disease (Jodeiri Farshbaf and Ghaedi, 2017)], and myopathy (Kim, 2017; Xie et al., 2020; Andrieux et al., 2021). Recently, the bioinformatics-based construction of prognostic signatures to predict the prognosis and guide treatment for patients with the terminal stage of malignant tumors has become a research hotspot (Chen et al., 2021a; Chen et al., 2021b; Zhang et al., 2024). Zhuo et al. (2021) established a mitophagy-related signature to explore the survival outcomes, tumor immunity, mutation, and chemotherapy response in pancreatic cancer. In addition, Zhang et al. (2021) constructed a mitochondria-related signature to explore the TIME, infiltration of immune cells, and immunotherapy of patients with hepatocellular carcinoma.

In the present study, we identified DEmrlncRNAs related to prognosis, which were utilized for subsequent consensus cluster analysis and prognostic signature construction. The patients with LUAD were classified into three clusters, and patients in cluster 1 exhibited the best survival outcomes. The patients in cluster 3 had the highest expression of *PDL1* and *HAVCR2*, and the highest TMB, which indicated that they might be the most sensitive to immunotherapy. The patients in cluster 2 had the lowest StromalScore, ImmuneScore, and ESTIMATEScore. According to the risk assessment model, patients in the low-risk group tended to have a significantly better survival outcome than the patients in the other groups. Furthermore, the risk score and stage could act as reliable independent prognostic indicators for patients with LUAD. The prognostic signature exhibited an excellent predictive ability among patients without distant metastasis, regardless of age, sex, stage, T, and N. The clinical heatmap revealed that the prognostic signature was closely related to N, T, stage, ImmuneScore, and molecular subtypes.

The prognostic signature is a novel and effective biomarker to select anti-tumor drugs. The low-risk patients tended to have a higher expression of *CTLA4* and *HAVCR2*, with a lower TMB. According to the IPS value, the low-risk patients would always benefit from anti-PD-L1 therapy, anti-*CTLA4* therapy, and their combination, which was significantly different. Furthermore, compared with the high-risk patients, the patients in the low-risk group tended to have a stronger tumor immunity. Furthermore, the risk assessment model could function as a robust guideline to select clinical medication comprising common chemotherapy and molecular targeted therapy. For example, patients in the high-risk group were more sensitive to Cisplatin, Docetaxel, Erlotinib, Gemcitabine, and Paclitaxel, while the low-risk patients would probably benefit more from Gefitinib. The high-risk patients in cluster 2 and cluster 3 exhibited poor survival outcomes, while the low-risk patients in cluster 1 showed a relative survival advantage.

The present study was the first to carry out bioinformatic analyses closely related to mitochondria and patients with LUAD. Furthermore, compared with traditional modeling process, we established a novel mitochondria-related algorithm containing a consensus cluster analyses and risk assessment model, in which patients with LUAD were classified into six groups that received different treatment strategies based on the corresponding groups. Moreover, least absolute shrinkage and selection operator regression analysis was carried out together with a multivariate Cox regression analysis to avoid overfitting of the model.

Although the algorithm might function as a guideline for clinical medication, there are also several limitations. Firstly, we conducted the internal verification using The Cancer Genome Atlas database, rather than other databases (e.g., GEO datasets). Secondly, all analyses were confined to bioinformatic analyses, and the study lacks validation of clinical specimens and molecular biological experiments, which are necessary for the clinical application of this algorithm.

Finally, based on the results of this manuscript, we have provided prospects for future potential research directions in this field. In recent years, the additional molecular pathways and incorporating multi-omics data integrations may play an increasingly important role in exploring the potential mechanisms of LUAD progression and treatment response. Nowadays, more and more research focuses on transcriptome sequencing. For example, studies based on transcriptome sequencing have demonstrated through *in vitro* and *in vivo* functional and mechanistic experiments that B4GALT1 promotes immune escape at both transcriptional and post transcriptional levels, thereby promoting the progression of LUAD (Cui et al., 2023). In addition, there are also studies based on single-cell RNA sequencing analysis data, which calculate the immunogenic cell death value of cells to construct a set of prognostic models that can predict the prognosis of LUAD patients and immunotherapy, and to some extent guide the clinical treatment of LUAD patients (Zhang et al., 2023a). More interestingly, researchers explored the role of Treg cells in Esophageal squamous cell carcinoma by combining single-cell RNA sequencing and bulk RNA-seq analysis, in order to predict patient prognosis and immune therapy responsiveness as a prognostic model (Zhang et al., 2023b).

In the present study, we constructed a novel and reliable algorithm comprising a consensus cluster analysis and risk assessment model to predict the survival outcomes, which function as a reliable guideline to select anti-tumor drugs to treat patients with terminal LUAD, which might provide a theoretical foundation for customized individualized treatment.

## Data Availability

The datasets presented in this study can be found in online repositories. The names of the repository/repositories and accession number(s) can be found in the article/supplementary material.

## Abbreviations

LUAD, Lung Adenocarcinoma; PDL1, Drogrammed cell Death Ligand 1; HAVCR2, Hepatitis A Virus Cellular Receptor 2; TMB, tumor mutation burden; CTLA4, Cytotoxic T-Lymphocyte Associated protein 4; OS, overall survival; TIME, Tumor Immune Microenvironment; ICIs, Immune Checkpoint Inhibitors; lncRNAs, Long Noncoding RNAs; GTF, Gene Transfer Format; GO, Gene Ontology; mrRNA, Mitochondria-Related lncRNAs; DEmrlncRNAs, Differentially Expressed mrlncRNAs; IPS, Immunophenoscore; IC_50_, half maximal Inhibitory Concentration; CDF, Cumulative Distribution Function.
